# Multi-year study monitoring the mercury content in the tissues of cattle sampled in the Czech Republic between 2014 and 2023

**DOI:** 10.17221/34/2025-VETMED

**Published:** 2025-09-26

**Authors:** Martin Svoboda, Veronika Vlasakova, Danka Harustiakova, Josef Illek, Andrea Staffa, Jan Vasek, Kamila Novotna Kruzikova, Jana Cahova, Zdenka Svobodova

**Affiliations:** ^1^Ruminant and Swine Clinic, Faculty of Veterinary Medicine, University of Veterinary Sciences Brno, Brno, Czech Republic; ^2^Central Veterinary Administration of the State Veterinary Administration, Prague, Czech Republic; ^3^RECETOX, Faculty of Science, Masaryk University, Brno, Czech Republic; ^4^Large Animal Clinical Laboratory, Faculty of Veterinary Medicine, University of Veterinary Sciences Brno, Brno, Czech Republic; ^5^Department of Animal Protection and Welfare and Veterinary Public Health, Faculty of Veterinary Hygiene and Ecology, University of Veterinary Sciences Brno, Brno, Czech Republic

**Keywords:** calf, cow, heavy metal, kidney, liver

## Abstract

Analyses of mercury concentrations in the muscle, liver and kidneys of cattle were conducted in the Czech Republic during the period from 2014 to 2023. The average mercury content in muscles, livers, and kidneys of calves was 0.000 5 ± 0.000 0 mg.kg^–1^, 0.002 7 ± 0.000 5 mg.kg^–1^, and 0.004 1 ± 0.000 8 mg.kg^–1^, respectively. In fattening cattle, the average mercury content in muscles, livers and kidneys was 0.000 5 ± 0.000 0 mg.kg^–1^, 0.002 1 ± 0.000 2 mg.kg^–1^ and 0.004 9 ± 0.000 3 mg.kg^–1^, respectively. In cows, the average mercury content in muscles, livers and kidneys was 0.000 5 ± 0.000 0 mg.kg^–1^, 0.002 3 ± 0.000 1 mg.kg^–1^ and 0.006 9 ± 0.000 3 mg.kg^–1^, respectively. The maximum residual limit for human consumption was exceeded in 10 kidney samples (3 calves, 6 cows, 1 fattening cattle) and 1 liver sample (calf). In all age categories, the highest mercury concentrations were found in the kidneys, lower in the livers, and the lowest in the muscles. When comparing the age groups, significantly higher mercury concentrations were observed in the kidneys of cows than in calves and fattening cattle. It can be concluded that there is still a need for further monitoring of mercury concentrations in cattle tissues in the Czech Republic.

Mercury is a toxic environmental contaminant. It does not degrade in the environment and becomes mobile due to its volatility. It can be transported by air over very long distances (Kabata-Pendias and Pendias 2001).

Both natural and anthropogenic sources contribute to environmental contamination with this element (Gworek et al. 2020). Mercury is naturally released by forest fires, volcanic activity, and the weathering of rocks (Sundseth et al. 2017).

Between 25% and 30% of the total amount of mercury in the atmosphere originates from anthropogenic sources (Eisler 1987). These include the combustion of fossil fuels and various industrial activities (Gworek et al. 2020).

Mercury in contaminated soil can enter the food chain through plants and livestock (Clifton 2007). It can bioaccumulate and cause adverse effects in both animals and humans (Pathak and Bhowmik 1998; Rice et al. 2014).

Animals can be exposed to various forms of mercury throughout their lifetimes. Possible routes of mercury exposure in animals include inhalation of mercury vapor from the air and ingestion through contaminated water and feed (Pirrone et al. 2001).

Ruminants are primarily exposed to mercury contamination through pasture feeding in summer and hay feeding in winter. Mercury enters grasses mainly from the soil (Rudy 2009).

According to Rudy (2009), mercury concentrations in tissues of cattle accurately reflect environmental levels. Therefore, cattle can be used as suitable indicator organisms for monitoring environmental contamination.

Studies investigating mercury content in livestock tissues suggest that cattle may contribute to human mercury intake (Rudy 2009; Lopez-Alonso et al. 2017).

The present study aimed to analyse and present the results of mercury content in bovine tissues as a part of a state monitoring programme conducted in the Czech Republic from 2014 to 2023.

## MATERIAL AND METHODS

### Sampling and analyses

Inspectors from the State Veterinary Admin-istration conducted tissue sampling from randomly selected animals in slaughterhouses across the Czech Republic. The aim was to achieve the broadest possible geographical coverage.

This sampling was part of the national residue control plan, which was conducted following the Council Directive 96/23/EC until 2022 and, since 2023, has been conducted under the new EU rules – specifically Commission Delegated Regulation (EU) 2022/931 and Commission Implementing Regulation (EU) 2022/932. The maximum residual limit (MRL) for human consumption is 0.01 mg.kg^–1^ for mercury in muscles and 0.02 mg.kg^–1^ for mercury in liver and kidneys. These values are given in the European Commission Regulation No. 2018/73 (European Commission 2018).

Samples of muscle (lean meat), any part of the liver (minimum 0.5 kg), and the whole kidneys were collected for analyses of total mercury content.

The analyses were performed in the laboratories of the State Veterinary Institutes in the Czech Republic using validated methods.

All laboratories are accredited according to EN ISO/IEC 17025:2018 and regularly participate in proficiency testing.

The total mercury content in cattle tissues was determined by the cold vapour atomic absorption spectrometry on an AMA 254 analyser (Altec Ltd., Dvůr Králové nad Labem, Czech Republic). The limit of quantification (LOQ) (at a sample weight of 50 mg) for muscle, liver, and kidneys was 0.000 5 mg.kg^–1^. Values below the LOQ were replaced by half of this limit, i.e., by the value of 0.000 2 mg.kg^–1^.

Analytical quality was ensured through the use of reference, duplicate, and blank samples. The certificate reference material (CRM) NIST 1566b Oyster Tissue is used (NIST: National Institute of Standards and Technology, Gaithersburg, MD 20899, USA).

Certified content of total mercury is 0.037 1 ± 0.001 3 mg.kg^–1^. This CRM is regularly analysed together with the measured samples, and the recovery is in the range of 90% to 100%.

### Statistical analysis

A total of 511 cattle were examined from 2014 to 2023, including 71 calves, 282 cows, and 158 fattening cattle. The measured mercury content values were compared with the maximum residual limit (MRL) for human consumption, which is 0.01 mg.kg^–1^ for mercury in muscles and 0.02 mg.kg^–1^ for mercury in liver and kidneys (European Commission 2018). The number of samples in individual years ranged from 3 to 54. Since the mercury content had a log-normal distribution, the values were logarithmically transformed before statistical processing. Year-to-year comparisons of values (separately for individual tissues and age groups) were performed using Kruskal-Wallis ANOVA and did not show significant differences (*P* > 0.05 in all comparisons). Furthermore, no statistically significant trend of a decrease in mercury content in tissues was measured during the observed period (Pearson’s correlation between the mercury concentration and year was very weak, from –0.085 to –0.179). Therefore, all data were processed together regardless of the year of sampling. Differences in mercury content among liver, kidney, and muscle tissues and animal groups were analysed using the repeated measures ANOVA with the animal group as a categorical factor and tissue as the repeated measures factor (since the values in the three tissues were always measured in the same individual). The analysis was performed on logarithmically transformed data, and homogeneity of variances was verified by Bartlett’s test. Repeated measures ANOVA was followed by Fisher’s post hoc test to identify differences between sample groups. A *P*-value of <0.05 was considered significant in all tests. Data processing and statistical analysis were performed using Statistica v14 (TIBCO Software Inc., San Ramon, USA).

## RESULTS

Mercury content in muscles ranged from 0.000 2 to 0.006 0 mg.kg^–1^ and did not exceed the maximum residual limit (MRL: 0.01 mg.kg^–1^) for human consumption in any sample. Mercury concentration in kidneys ranged from 0.000 2 to 0.051 9 mg.kg^–1^ and in livers from 0.000 2 to 0.025 1 mg.kg^–1^. The MRL for human consumption (0.02 mg.kg^–1^) was exceeded in 10 kidney samples (3 calves, 6 cows, 1 fattening cattle) and one liver sample (calf) (Table 1).

**Table 1:** Mercury content in tissues of cattle (mg.kg^–1^)

Age category	Tissue	*N*	Median	Mean ± SE	Min–max
Calves	muscle	71	0.000 5	0.000 5 ± 0.000 0^A,a^	0.000 2–0.002 7
	kidney	71	0.002 0	0.004 1 ± 0.000 8^B,a^	0.000 2–0.040 0
	liver	71	0.001 2	0.002 7 ± 0.000 5^C,a^	0.000 2–0.025 1
Cows	muscle	282	0.000 5	0.000 5 ± 0.000 0^A,a^	0.000 2–0.006 0
	kidney	280	0.005 4	0.006 9 ± 0.000 3^B,b^	0.000 2–0.051 9
	liver	282	0.001 7	0.002 3 ± 0.000 1^C,a^	0.000 2–0.012 1
Fattening cattle	muscle	158	0.000 5	0.000 5 ± 0.000 0^A,a^	0.000 2–0.003 4
	kidney	158	0.004 0	0.004 9 ± 0.000 3^B,c^	0.001 0–0.041 7
	liver	158	0.001 6	0.002 1 ± 0.000 2^C,a^	0.000 2–0.012 6

^A–C^The mercury concentration in different tissues followed by the same uppercase superscript did not differ significantly (separately for calves, cows and fattening cattle); ^a,b^The mercury concentration in different groups of animals followed by the same lowercase superscript did not differ significantly (separately for liver, kidney and muscle)

Mercury concentration was significantly influenced by tissue type (repeated measures ANOVA: the effect of tissue: F(2,1012) = 1 333.962, *P* < 0.001) as well as by animal group (repeated measures ANOVA: effect of age group: F(2,506) = 9.752, *P* < 0.001). A detailed comparison revealed significant differences between tissue types among calves, cows, and fattening cattle. In calves, the highest mercury content was found in the kidneys (0.004 1 ± 0.000 8 mg.kg^–1^), followed by the liver (0.002 7 ± 0.000 5 mg.kg^–1^), and the lowest levels in the muscles (0.000 5 ± 0.000 0 mg.kg^–1^). Differences among all three tissues were statistically significant (*P* < 0.05 for all comparisons). A similar pattern was observed in cows with the highest mercury content in the kidneys (0.006 9 ± 0.000 3 mg.kg^–1^), lower in the liver (0.002 3 ± 0.000 1 mg.kg^–1^), and the lowest in the muscles (0.000 5 ± 0.000 0 mg.kg^–1^); again, all differences were significant (*P* < 0.05 for all comparisons). In fattening cattle, the same trend was found: kidneys (0.004 9 ± 0.000 3 mg.kg^–1^), lower in the liver (0.002 1 ± 0.000 2 mg.kg^–1^) and the lowest in the muscles (0.000 5 ± 0.000 0 mg.kg^–1^) with significant differences among all tissues (*P* < 0.05; Table 1).

When comparing the age groups, cows had significantly higher mercury levels in kidneys than both calves and fattening cattle, while significant differences were also found between calves and fattening cattle (*P* < 0.05 for all three comparisons; Table 1).

For muscles and liver, no significant differences in mercury concentrations were observed among the age groups of cattle (*P* > 0.05 for all comparisons; Table 1).

## DISCUSSION

Environmental mercury can exist in its elemental, inorganic, or organic forms. These forms may be emitted into the atmosphere and transported over very long distances (Gworek et al. 2020).

On land surfaces, mercury is deposited mainly in its oxidised form (Hg^2+^). Its further transformation depends on the redox potential of the environment. Inorganic forms Hg^2+^ and Hg^+^ occur in soils under oxidising conditions, while elemental mercury and sulphur compounds are found mainly in reducing conditions. Organic methylmercury compounds can be found in soils with transitional conditions (Selin et al. 2018).

Both inorganic and organic mercury compounds can be present in animal tissues. Inorganic mercury salts are not fat-soluble and therefore do not cross the blood-brain barrier or the placental barrier (Rice et al. 2014). The most important organic form is methylmercury, which is more toxic than inorganic forms due to its lipophilicity, allowing it to easily cross cell membranes, including the placental and blood–brain barriers (Clarkson et al. 2007).

It was beyond the scope of our study to differentiate between mercury species in the analysed tissues. Although chromatographic techniques are now available to distinguish between inorganic and organic mercury, these methods are complex and expensive, and therefore not routinely used (EFSA 2008). For these reasons, only total mercury content was measured in our study.

Generally, both inorganic and organic mercury compounds show a similar distribution pattern in animal tissues – the highest concentrations are found in kidneys and liver, while the lowest levels are present in muscle tissue (Gyrd-Hansen 1981; Lohren et al. 2015). This is consistent with the findings of our study.

In our study, mercury concentrations were significantly higher in the kidneys than in the liver, which agrees with previous studies on cattle (Niemi et al. 1991; Salisbury et al. 1991). This may be due to the ability of inorganic mercury to bind to metallothionein in the kidneys (Goyer 1995; Simpson et al. 1997). In contrast, elemental mercury intake does not induce metallothionein synthesis in the liver (Goyer 1995).

In our study, we also found significantly higher mercury concentrations in the kidneys of cows compared to calves and fattening cattle. This could be explained by the tendency of mercury to accumulate in the kidneys, which increases with the age of the animals (Clarkson et al. 2007). Fish are an exception to this claim. In case of the uncontaminated or slightly contaminated sites, the mercury content in the muscles of fish is usually higher than in the liver (Novotna Kruzikova et al. 2023).

However, other studies have reported different trends. For instance, Vos et al. (1987) and Lopez-Alonso et al. (2003) found that kidney mercury concentrations tended to be higher in younger cattle than in older animals. Oskarsson et al. (1998) explained this fact by the higher relative food intake, greater gastrointestinal absorption, and less efficient renal excretion of mercury in comparison to adults. We assume these factors played a less significant role in our study.

Below, we compare our results with other studies conducted in Europe. In Poland, Nawrocka et al. (2020) detected total mercury levels in muscle (0.000 8 ± 0.001 2 mg·kg^–1^) and liver (0.0020 ± 0.0022 mg·kg^–1^). They found that muscle mercury concentrations remained stable at trace levels over the study period. They noted a decline in mercury bioaccumulation in the liver of cattle in Poland from 2009 to 2018, reflecting a general decrease in mercury emissions across Europe in recent decades.

In Serbia, Tomovic et al. (2021) analysed mercury concentrations in the liver and kidney tissues of 26 dairy cows (age range: 412–2 502 days) in the Vojvodina region. Liver concentrations ranged from below the detection limit (LOD < 0.006 mg·kg^–1^) to a maximum of 0.206 mg·kg^–1^ wet weight, while the concentrations in the kidney varied from below the detection limit (LOD < 0.006 mg·kg^–1^) to a peak value of 0.018 mg·kg^–1^ wet weight.

Another study measured mercury concentrations in the liver, kidneys, and meat of calves in Spain (Lopez-Alonso et al. 2003). In their study, mercury was detected in 62.4–87.5% of kidney samples, while most liver, muscle, and blood samples (79.5–96%) showed no detectable residues. Kidney mercury concentrations were significantly higher in calves from the predominantly rural region of Galicia (geometric mean: 12.2 μg/kg w.wt.) than those from the industrialised-mining region of Asturias (3.40 μg/kg w.wt.). Kocasari et al. (2017) examined the content of heavy metals in muscle, liver, and kidney tissues of cattle from Burdur, Turkey, between April and June 2014. Samples were collected from 50 slaughtered cattle. The study found that mercury was not detected in any of the samples, suggesting a low level of environmental contamination by this metal in the region.

In the Iranian study, Hashemi (2018) measured mercury (Hg) concentrations in bovine tissues from 72 cows, with a total of 216 samples (muscle, liver, and kidney) collected across four seasons in Fars province, Iran. The concentrations of mercury (mg.kg^–1^ wet weight) in the tissues were as follows: muscle 0.003, liver 0.002, and kidney 0.003. All mercury concentrations were below the European Union’s maximum residual limits (MRL), indicating they were within safe limits for human consumption. Additionally, no explicit correlation was observed between mercury levels in the feed and those found in the bovine tissues.

In the Czech Republic, Drapal and Haldova (2014) observed equal mercury concentrations in the liver and kidneys of calves (median: 0.002 0 mg·kg^–1^). In bulls and heifers (up to 2 years of age), the highest mercury concentrations were recorded in kidneys (median 0.005 9 mg·kg^–1^), followed by liver (median 0.002 4 mg·kg^–1^). In dairy cows, kidney mercury levels were highest (median: 0.007 4 mg·kg^–1^), with lower concentrations in liver (median 0.002 8 mg·kg^–1^) and the lowest in muscle (median 0.000 5 mg·kg^–1^).

The total mercury content in the liver and, particularly, in the kidneys of cattle increased with the age of the animals, which aligns with the data presented in our study. Furthermore, our data show a general decline in mercury concentrations in both the liver and kidneys across all categories of cattle in the Czech Republic during the 2014–2023 period, when compared to the 2010–2013 period (Drapal and Haldova 2014).

Differences in reported mercury concentrations across studies can be attributed to regional differences in environmental contamination and feed composition (Barghigiani and Ristori 1994).

The mercury concentrations detected in our study were significantly below levels associated with mercury-induced renal dysfunction (Simpson et al. 1997; Pathak and Bhowmik 1998). Therefore, environmental mercury contamination is unlikely to pose a health risk to cattle in the Czech Republic.

The main motivation for determining mercury content in cattle tissues in our study was to assess the potential health risk to humans consuming cattle-derived products.

The maximum residual limit (MRL) for human consumption (0.01 mg.kg^–1^ for mercury in muscles and 0.02 mg.kg^–1^ for mercury in liver and kidneys; European Commission 2018) was exceeded in 10 kidney samples (3 calves, 6 cows, 1 fattening cattle) and one liver sample (calf).

No statistically significant trend in the decrease of mercury levels in cattle tissues was found during the study period. We therefore conclude that continued monitoring of mercury concentrations in cattle tissues is warranted in the Czech Republic.

## References

[R1] Barghigiani C, Ristori T. Mercury levels in agricultural products of Mt. Amiata (Tuscany, Italy). Arch Environ Contam Toxicol. 1994 Apr;26(3):329-34. 8161231 10.1007/BF00203559

[R2] Clarkson TW, Vyas JB, Ballatori N. Mechanisms of mercury disposition in the body. Am J Ind Med. 2007 Oct;50(10): 757-64. 17477364 10.1002/ajim.20476

[R3] Clifton JC 2nd. Mercury exposure and public health. Pediatr Clin North Am. 2007 Apr;54(2):237-69, viii. 17448359 10.1016/j.pcl.2007.02.005

[R4] Drapal J, Haldova S. Obsah rtuti v mase a organech skotu a prasat [Mercury content in the meat and organs of cattle and pigs]. In: Zborník prednášok a posterov; 2014 May 21–23; Štrbské Pleso, Slovakia. Maso International. 2014;4:87-90. Czech.

[R5] European Commission. Commission regulation (EU) 2018/73 of 16 January 2018 amending Annexes II and III to Regulation (EC) No. 396/2005 of the European Parliament and of the Council as regards maximum residue levels for mercury compounds in or on certain products [Internet]. 2018 [cit. 2025 Jan 30]. Available: https://eur-lex.europa.eu/eli/reg/2018/73/oj/eng.

[R6] EFSA – European Food Safety Authority. Mercury as undesirable substance in feed. Opinion of the Scientific Panel on Contaminants in the food chain. EFSA J. 2008 Apr;654:1-76.

[R7] Eisler R. Mercury hazards to fish, wildlife and invertebrates: a synoptic review. Laurel (MD): U.S. Fish and Wildlife Service; 1987. 63 p. Report No.: 85(1.10).

[R8] Goyer RA. Factors influencing metal toxicity. In: Goyer RA, Klaasse CD, Waalkes MP, editors. Metal toxicology. San Diego, USA: Academic Press Inc; 1995. p. 31-45.

[R9] Gworek B, Dmuchowski W, Baczewska-Dabrowska AH. Mercury in the terrestrial environment: A review. Environ Sci Eur. 2020 Oct;32:128.

[R10] Gyrd-Hansen N. Toxicokinetics and methyl mercury in pigs. Arch Toxicol. 1981 Sep;48(2-3):173-81. 7295033 10.1007/BF00310486

[R11] Kocasari F, Yurdakul OK, Kart A, Yalcin H, Keyvan, Bayezit M. Few heavy metal levels in certain tissues of cattle in Burdur of Turkey. Indian J Anim Res. 2017 Aug;51(4): 759-61.

[R12] Hashemi M. Heavy metal concentrations in bovine tissues (muscle, liver and kidney) and their relationship with heavy metal contents in consumed feed. Ecotoxicol Environ Saf. 2018 Jun 15;154:263-7. 29476976 10.1016/j.ecoenv.2018.02.058

[R13] Kabata-Pendias A, Pendias H. Trace elements in soils and plants. 3^rd^ ed. Boca Raton, Florida, USA: CRC Press LLC; 2001. 403 p.

[R14] Lohren H, Blagojevic L, Fitkau R, Ebert F, Schildknecht S, Leist M, Schwerdtle T. Toxicity of organic and inorganic mercury species in differentiated human neurons and human astrocytes. J Trace Elem Med Biol. 2015 Oct;32: 200-8. 26302930 10.1016/j.jtemb.2015.06.008

[R15] Lopez-Alonso M, Benedito JL, Miranda M, Castillo C, Hernandez J, Shore RF. Mercury concentrations in cattle from NW Spain. Sci Total Environ. 2003 Jan 20;302(1-3):93-100. 12526901 10.1016/s0048-9697(02)00345-5

[R16] Lopez-Alonso M, Rey-Crespo F, Herrero-Latorre C, Miranda M. Identifying sources of metal exposure in organic and conventional dairy farming. Chemosphere. 2017 Oct;185:1048-55. 28764100 10.1016/j.chemosphere.2017.07.112

[R17] Nawrocka A, Durkalec M, Szkoda J, Filipek A, Kmiecik M, Zmudzki J, Posyniak A. Total mercury levels in the muscle and liver of livestock and game animals in Poland, 2009–2018. Chemosphere. 2020 Nov;258:127311. 32540547 10.1016/j.chemosphere.2020.127311

[R18] Niemi A, Venalainen ER, Hirvi T, Hirn J, Karppanen E. The lead, cadmium and mercury concentrations in muscle, liver and kidney from Finnish pigs and cattle during 1987–1988. Z Lebensm Unters Forsch. 1991 May;192(5): 427-9. 2058312 10.1007/BF01193141

[R19] Novotna Kruzikova K, Siroka Z, Svobodova Z. Total mercury content in selected tissues of common carp (Cyprinus carpio) pond farmed in the Czech Republic. Acta Veterinaria Brno. 2023 Dec;92(4):419-25.

[R20] Oskarsson A, Hallen IP, Sundberg J, Grawe KP. Risk assessment in relation to neonatal metal exposure. Analyst. 1998 Jan;123(1):19-23. 9581014 10.1039/a705136k

[R21] Pathak SK, Bhowmik MK. The chronic toxicity of inorganic mercury in goats: Clinical signs, toxicopathological changes and residual concentrations. Vet Res Commun. 1998 Feb;22(2):131-8. 9563171 10.1023/a:1006031630434

[R22] Pirrone N, Costa P, Pacyna JM, Ferrara R. Mercury emissions to the atmosphere from natural andanthropogenic sources in the Mediterranean region. Atmos Environ. 2001 Jun;35(17):2997-3006.

[R23] Rice KM, Walker EM, Wu M, Gillette C, Blough ER. Environmental mercury and its toxic effects. J Prev Med Public Health. 2014 Mar;47(2):74-83. 24744824 10.3961/jpmph.2014.47.2.74 PMC3988285

[R24] Rudy M. Correlation of lead, cadmium and mercury levels in tissue and liver samples with age in cattle. Food Addit Contam Part A Chem Anal Control Expo Risk Assess. 2009 Jun;26(6):847-53. 19680959 10.1080/02652030902835747

[R25] Salisbury DC, Chan W, Saschenbrecker PW. Multielement concentrations in liver and kidney tissues from five species of Canadian slaughter animals. J Assoc Off Anal Chem. 1991 Jul-Aug;74(4):587-91. 1917804

[R26] Selin H, Keane SE, Wang S, Selin NE, Davis K, Bally D. Linking science and policy to support the implementation of the Minamata Convention on Mercury. Ambio. 2018 Mar;47(2):198-215. 29388129 10.1007/s13280-017-1003-x PMC5794682

[R27] Simpson VR, Stuart NC, Munro R, Hunt A, Livesey CT. Poisoning of dairy heifers by mercurous chloride. Vet Rec. 1997 May 24;140(21):549-52. 9185311 10.1136/vr.140.21.549

[R28] Sundseth K, Pacyna JM, Pacyna EG, Pirrone N, Thorne RJ. Global sources and pathways of mercury in the context of human health. Int J Environ Res Public Health. 2017 Jan 22;14(1):105. 28117743 10.3390/ijerph14010105 PMC5295355

[R29] Tomovic V, Jokanovic M, Tomovic M, Sojic B, Lazovic M, Vasiljevic I, Skaljac S, Martinovic A, Vujadinovic D, Vukic M. Mercury in female cattle livers and kidneys from Vojvodina, northern Serbia. IOP Conf. Ser.: Earth Environ Sci. 2021;854:012099.

[R30] Vos G, Hovens JP, Delft WV. Arsenic, cadmium, lead and mercury in meat, livers and kidneys of cattle slaughtered in the Netherlands during 1980–1985. Food Addit Contam. 1987 Jan-Mar;4(1):73-88. 3556678 10.1080/02652038709373617

